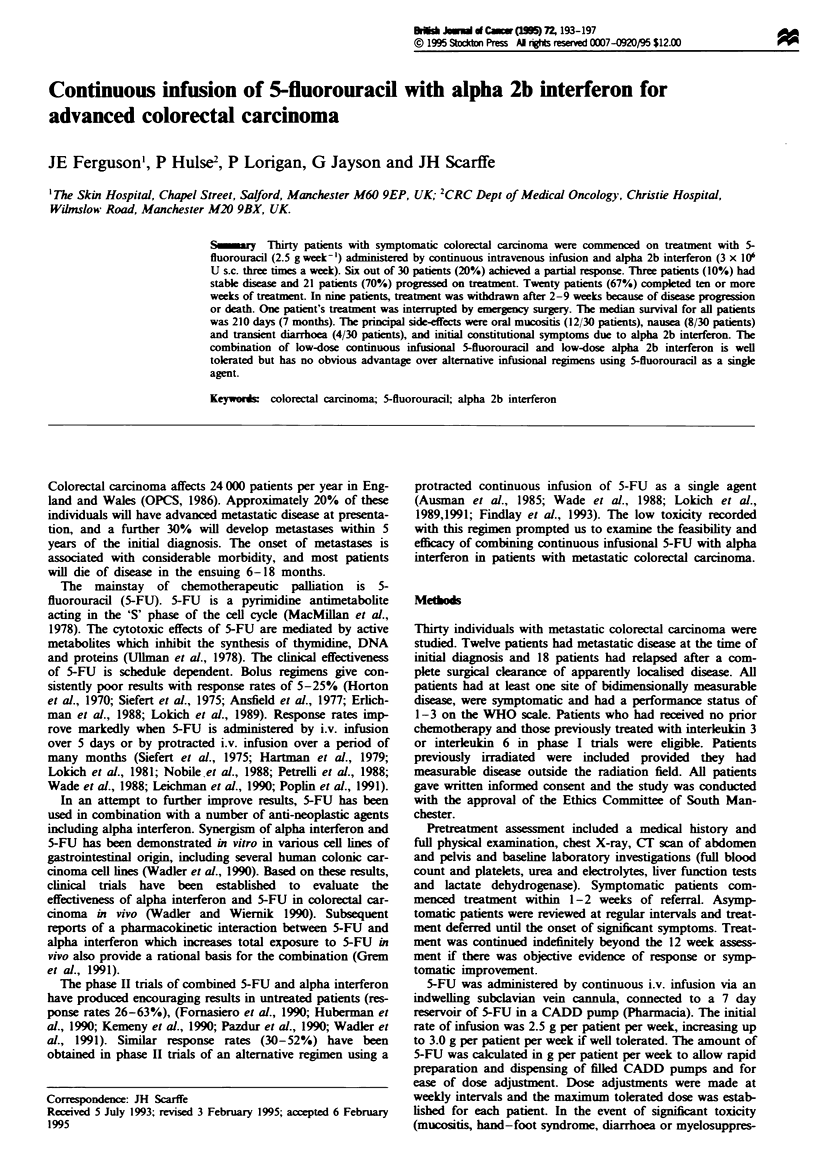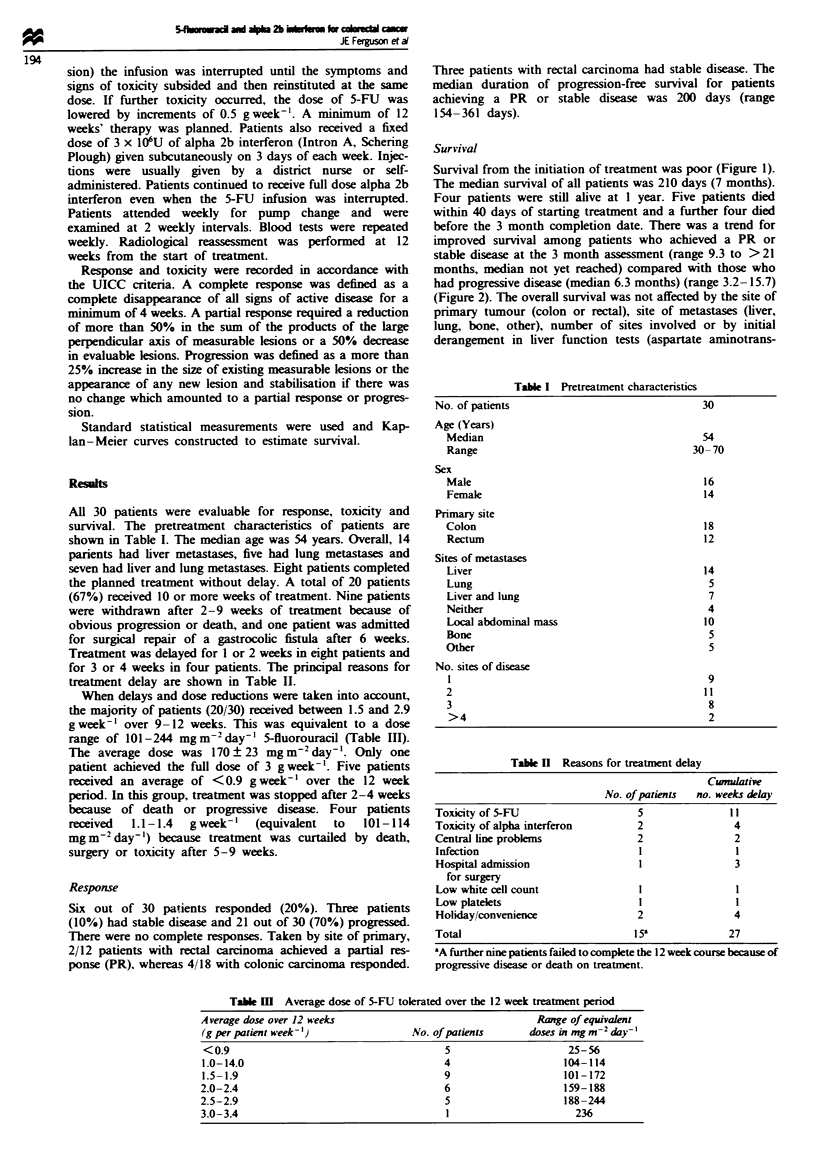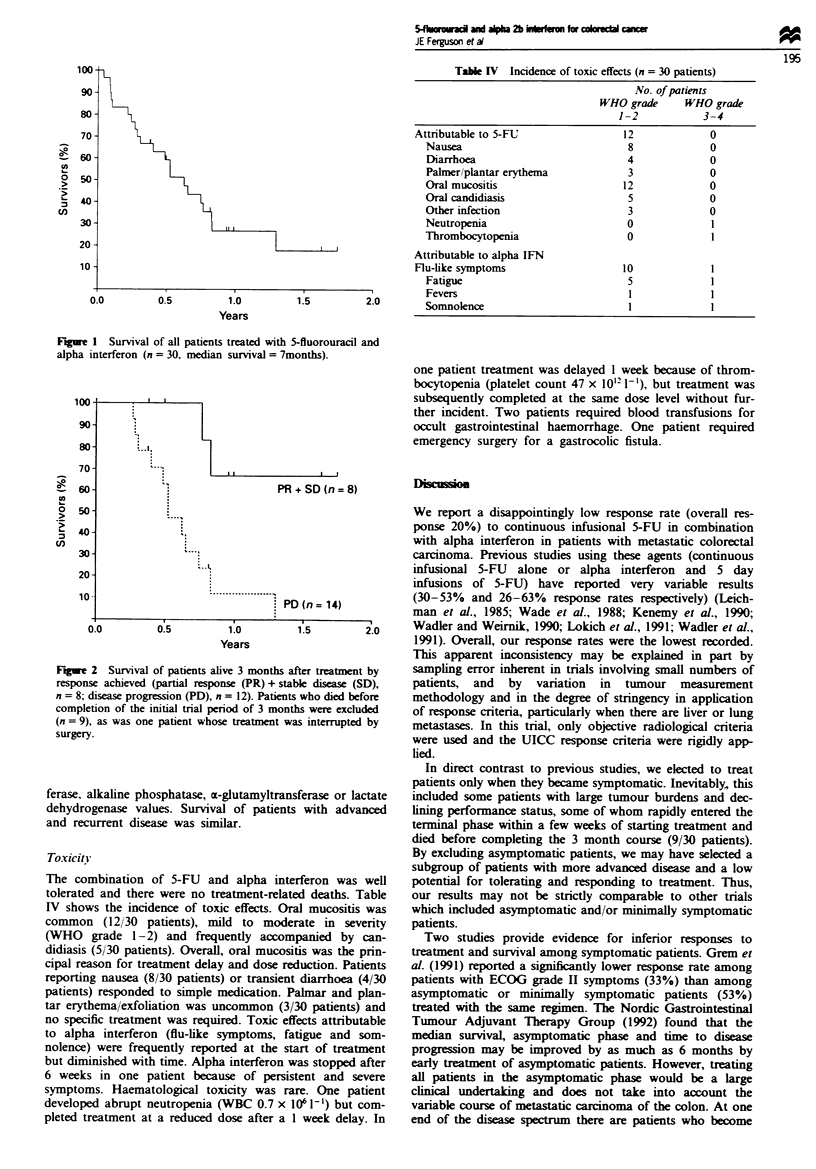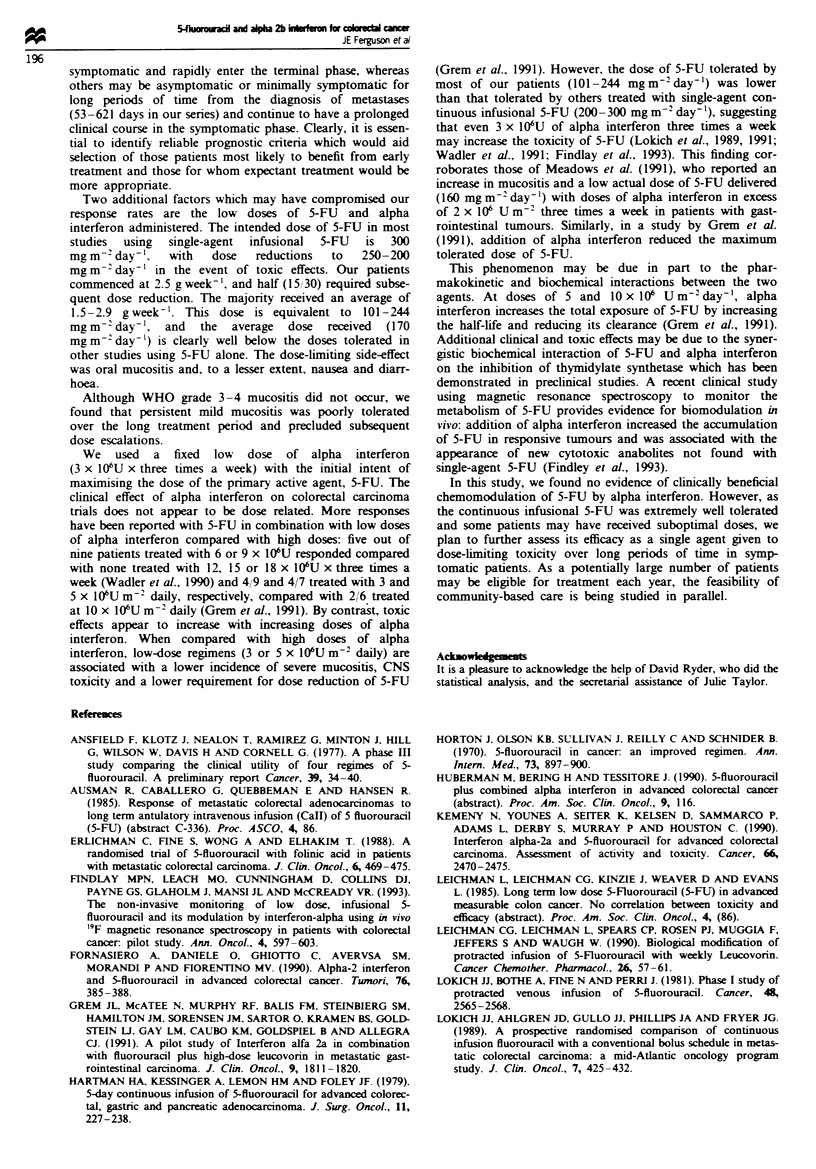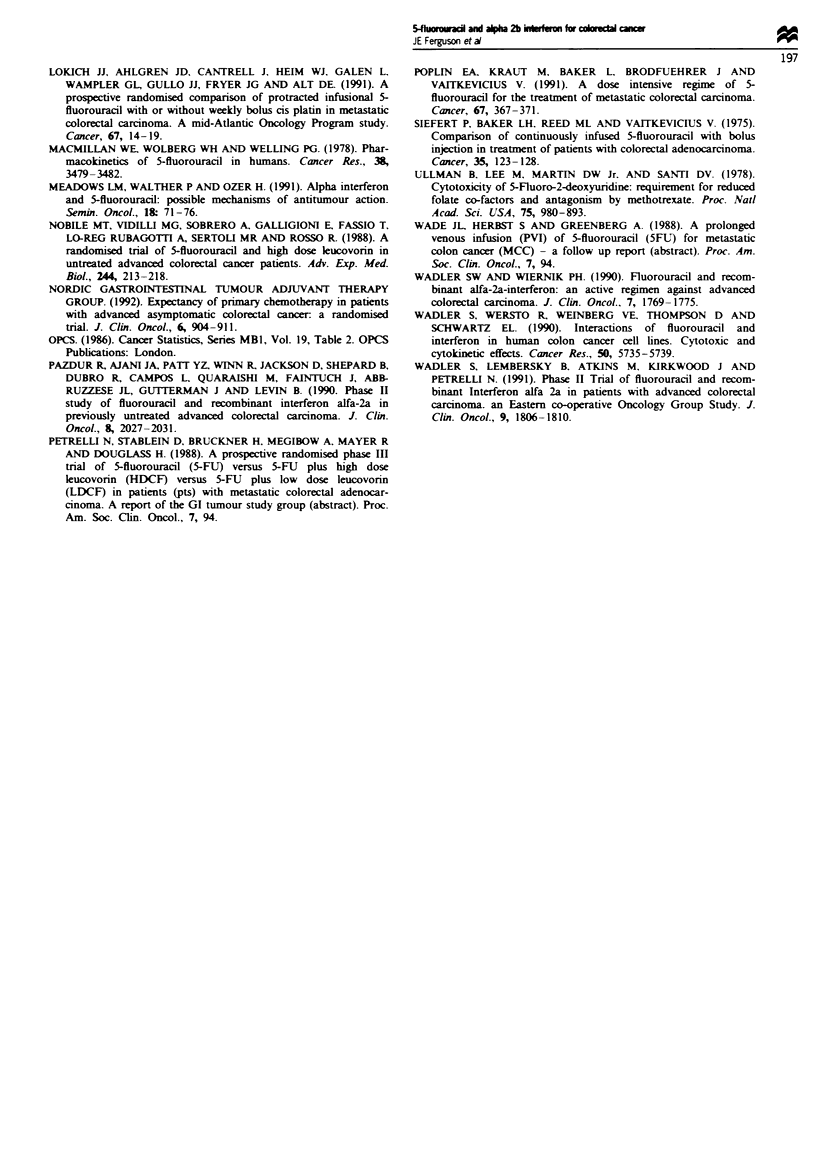# Continuous infusion of 5-fluorouracil with alpha 2b interferon for advanced colorectal carcinoma.

**DOI:** 10.1038/bjc.1995.302

**Published:** 1995-07

**Authors:** J. E. Ferguson, P. Hulse, P. Lorigan, G. Jayson, J. H. Scarffe

**Affiliations:** Skin Hospital, Salford, Manchester, UK.

## Abstract

Thirty patients with symptomatic colorectal carcinoma were commenced on treatment with 5-fluorouracil (2.5 g week-1) administered by continuous intravenous infusion and alpha 2b interferon (3 x 10(6) U s.c. three times a week). Six out of 30 patients (20%) achieved a partial response. Three patients (10%) had stable disease and 21 patients (70%) progressed on treatment. Twenty patients (67%) completed ten or more weeks of treatment. In nine patients, treatment was withdrawn after 2-9 weeks because of disease progression or death. One patient's treatment was interrupted by emergency surgery. The median survival for all patients was 210 days (7 months). The principal side-effects were oral mucositis (12/30 patients), nausea (8/30 patients) and transient diarrhoea (4/30 patients), and initial constitutional symptoms due to alpha 2b interferon. The combination of low-dose continuous infusional 5-fluorouracil and low-dose alpha 2b interferon is well tolerated but has no obvious advantage over alternative infusional regimens using 5-fluorouracil as a single agent.


© 1995 Stockton Press All rights reserved 0007-0920/95 $12.00

### Continuous infusion of 5-fluorouracil with alpha 2b interferon for advanced colorectal carcinoma

JE Ferguson[1], P Hulse[2], P Lorigan, G Jayson and JH Scarffe

[1]*The Skin Hospital, Chapel Street, Salford, Manchester M60 9EP, UK;* [2]*CRC Dept of Medical Oncology, Christie Hospital, Wilmslow Road, Manchester M20 9BX, UK.*

**Summary** Thirty patients with symptomatic colorectal carcinoma were commenced on treatment with 5-fluorouracil (2.5 g week$^{-1}$) administered by continuous intravenous infusion and alpha 2b interferon ($3 \times 10^6$ U s.c. three times a week). Six out of 30 patients (20%) achieved a partial response. Three patients (10%) had stable disease and 21 patients (70%) progressed on treatment. Twenty patients (67%) completed ten or more weeks of treatment. In nine patients, treatment was withdrawn after 2-9 weeks because of disease progression or death. One patient's treatment was interrupted by emergency surgery. The median survival for all patients was 210 days (7 months). The principal side-effects were oral mucositis (12/30 patients), nausea (8/30 patients) and transient diarrhoea (4/30 patients), and initial constitutional symptoms due to alpha 2b interferon. The combination of low-dose continuous infusional 5-fluorouracil and low-dose alpha 2b interferon is well tolerated but has no obvious advantage over alternative infusional regimens using 5-fluorouracil as a single agent.

**Keywords:** colorectal carcinoma; 5-fluorouracil; alpha 2b interferon

Colorectal carcinoma affects 24 000 patients per year in England and Wales (OPCS, 1986). Approximately 20% of these individuals will have advanced metastatic disease at presentation, and a further 30% will develop metastases within 5 years of the initial diagnosis. The onset of metastases is associated with considerable morbidity, and most patients will die of disease in the ensuing 6-18 months.

The mainstay of chemotherapeutic palliation is 5-fluorouracil (5-FU). 5-FU is a pyrimidine antimetabolite acting in the 'S' phase of the cell cycle (MacMillan *et al.*, 1978). The cytotoxic effects of 5-FU are mediated by active metabolites which inhibit the synthesis of thymidine, DNA and proteins (Ullman *et al.*, 1978). The clinical effectiveness of 5-FU is schedule dependent. Bolus regimens give consistently poor results with response rates of 5-25% (Horton *et al.*, 1970; Siefert *et al.*, 1975; Ansfield *et al.*, 1977; Erlichman *et al.*, 1988; Lokich *et al.*, 1989). Response rates improve markedly when 5-FU is administered by i.v. infusion over 5 days or by protracted i.v. infusion over a period of many months (Siefert *et al.*, 1975; Hartman *et al.*, 1979; Lokich *et al.*, 1981; Nobile *et al.*, 1988; Petrelli *et al.*, 1988; Wade *et al.*, 1988; Leichman *et al.*, 1990; Poplin *et al.*, 1991).

In an attempt to further improve results, 5-FU has been used in combination with a number of anti-neoplastic agents including alpha interferon. Synergism of alpha interferon and 5-FU has been demonstrated *in vitro* in various cell lines of gastrointestinal origin, including several human colonic carcinoma cell lines (Wadler *et al.*, 1990). Based on these results, clinical trials have been established to evaluate the effectiveness of alpha interferon and 5-FU in colorectal carcinoma *in vivo* (Wadler and Wiernik 1990). Subsequent reports of a pharmacokinetic interaction between 5-FU and alpha interferon which increases total exposure to 5-FU *in vivo* also provide a rational basis for the combination (Grem *et al.*, 1991).

The phase II trials of combined 5-FU and alpha interferon have produced encouraging results in untreated patients (response rates 26-63%), (Fornasiero *et al.*, 1990; Huberman *et al.*, 1990; Kemeny *et al.*, 1990; Pazdur *et al.*, 1990; Wadler *et al.*, 1991). Similar response rates (30-52%) have been obtained in phase II trials of an alternative regimen using a protracted continuous infusion of 5-FU as a single agent (Ausman *et al.*, 1985; Wade *et al.*, 1988; Lokich *et al.*, 1989,1991; Findlay *et al.*, 1993). The low toxicity recorded with this regimen prompted us to examine the feasibility and efficacy of combining continuous infusional 5-FU with alpha interferon in patients with metastatic colorectal carcinoma.

Correspondence: JH Scarffe
Received 5 July 1993; revised 3 February 1995; accepted 6 February 1995

### Methods

Thirty individuals with metastatic colorectal carcinoma were studied. Twelve patients had metastatic disease at the time of initial diagnosis and 18 patients had relapsed after a complete surgical clearance of apparently localised disease. All patients had at least one site of bidimensionally measurable disease, were symptomatic and had a performance status of 1-3 on the WHO scale. Patients who had received no prior chemotherapy and those previously treated with interleukin 3 or interleukin 6 in phase I trials were eligible. Patients previously irradiated were included provided they had measurable disease outside the radiation field. All patients gave written informed consent and the study was conducted with the approval of the Ethics Committee of South Manchester.

Pretreatment assessment included a medical history and full physical examination, chest X-ray, CT scan of abdomen and pelvis and baseline laboratory investigations (full blood count and platelets, urea and electrolytes, liver function tests and lactate dehydrogenase). Symptomatic patients commenced treatment within 1-2 weeks of referral. Asymptomatic patients were reviewed at regular intervals and treatment deferred until the onset of significant symptoms. Treatment was continued indefinitely beyond the 12 week assessment if there was objective evidence of response or symptomatic improvement.

5-FU was administered by continuous i.v. infusion via an indwelling subclavian vein cannula, connected to a 7 day reservoir of 5-FU in a CADD pump (Pharmacia). The initial rate of infusion was 2.5 g per patient per week, increasing up to 3.0 g per patient per week if well tolerated. The amount of 5-FU was calculated in g per patient per week to allow rapid preparation and dispensing of filled CADD pumps and for ease of dose adjustment. Dose adjustments were made at weekly intervals and the maximum tolerated dose was established for each patient. In the event of significant toxicity (mucositis, hand-foot syndrome, diarrhoea or myelosuppression) the infusion was interrupted until the symptoms and signs of toxicity subsided and then reinstituted at the same dose. If further toxicity occurred, the dose of 5-FU was lowered by increments of 0.5 g $week^{-1}$. A minimum of 12 weeks' therapy was planned. Patients also received a fixed dose of $3 \times 10^6$U of alpha 2b interferon (Intron A, Schering Plough) given subcutaneously on 3 days of each week. Injections were usually given by a district nurse or self-administered. Patients continued to receive full dose alpha 2b interferon even when the 5-FU infusion was interrupted. Patients attended weekly for pump change and were examined at 2 weekly intervals. Blood tests were repeated weekly. Radiological reassessment was performed at 12 weeks from the start of treatment.

Response and toxicity were recorded in accordance with the UICC criteria. A complete response was defined as a complete disappearance of all signs of active disease for a minimum of 4 weeks. A partial response required a reduction of more than 50% in the sum of the products of the large perpendicular axis of measurable lesions or a 50% decrease in evaluable lesions. Progression was defined as a more than 25% increase in the size of existing measurable lesions or the appearance of any new lesion and stabilisation if there was no change which amounted to a partial response or progression.

Standard statistical measurements were used and Kaplan–Meier curves constructed to estimate survival.

### Results

All 30 patients were evaluable for response, toxicity and survival. The pretreatment characteristics of patients are shown in Table I. The median age was 54 years. Overall, 14 parients had liver metastases, five had lung metastases and seven had liver and lung metastases. Eight patients completed the planned treatment without delay. A total of 20 patients (67%) received 10 or more weeks of treatment. Nine patients were withdrawn after 2–9 weeks of treatment because of obvious progression or death, and one patient was admitted for surgical repair of a gastrocolic fistula after 6 weeks. Treatment was delayed for 1 or 2 weeks in eight patients and for 3 or 4 weeks in four patients. The principal reasons for treatment delay are shown in Table II.

When delays and dose reductions were taken into account, the majority of patients (20/30) received between 1.5 and 2.9 g $week^{-1}$ over 9–12 weeks. This was equivalent to a dose range of 101–244 mg $m^{-2}$ $day^{-1}$ 5-fluorouracil (Table III). The average dose was $170 \pm 23$ mg $m^{-2}$ $day^{-1}$. Only one patient achieved the full dose of 3 g $week^{-1}$. Five patients received an average of $<0.9$ g $week^{-1}$ over the 12 week period. In this group, treatment was stopped after 2–4 weeks because of death or progressive disease. Four patients received 1.1–1.4 g $week^{-1}$ (equivalent to 101–114 mg $m^{-2}$ $day^{-1}$) because treatment was curtailed by death, surgery or toxicity after 5–9 weeks.

### *Response*

Six out of 30 patients responded (20%). Three patients (10%) had stable disease and 21 out of 30 (70%) progressed. There were no complete responses. Taken by site of primary, 2/12 patients with rectal carcinoma achieved a partial response (PR), whereas 4/18 with colonic carcinoma responded. Three patients with rectal carcinoma had stable disease. The median duration of progression-free survival for patients achieving a PR or stable disease was 200 days (range 154–361 days).

### *Survival*

Survival from the initiation of treatment was poor (Figure 1). The median survival of all patients was 210 days (7 months). Four patients were still alive at 1 year. Five patients died within 40 days of starting treatment and a further four died before the 3 month completion date. There was a trend for improved survival among patients who achieved a PR or stable disease at the 3 month assessment (range 9.3 to >21 months, median not yet reached) compared with those who had progressive disease (median 6.3 months) (range 3.2–15.7) (Figure 2). The overall survival was not affected by the site of primary tumour (colon or rectal), site of metastases (liver, lung, bone, other), number of sites involved or by initial derangement in liver function tests (aspartate aminotrans-

**Table I** Pretreatment characteristics

| | |
|---|---|
| No. of patients | 30 |
| Age (Years) | |
| Median | 54 |
| Range | 30–70 |
| Sex | |
| Male | 16 |
| Female | 14 |
| Primary site | |
| Colon | 18 |
| Rectum | 12 |
| Sites of metastases | |
| Liver | 14 |
| Lung | 5 |
| Liver and lung | 7 |
| Neither | 4 |
| Local abdominal mass | 10 |
| Bone | 5 |
| Other | 5 |
| No. sites of disease | |
| 1 | 9 |
| 2 | 11 |
| 3 | 8 |
| >4 | 2 |

**Table II** Reasons for treatment delay

| | *No. of patients* | *Cumulative no. weeks delay* |
|---|---|---|
| Toxicity of 5-FU | 5 | 11 |
| Toxicity of alpha interferon | 2 | 4 |
| Central line problems | 2 | 2 |
| Infection | 1 | 1 |
| Hospital admission for surgery | 1 | 3 |
| Low white cell count | 1 | 1 |
| Low platelets | 1 | 1 |
| Holiday/convenience | 2 | 4 |
| Total | 15[a] | 27 |

[a]A further nine patients failed to complete the 12 week course because of progressive disease or death on treatment.

**Table III** Average dose of 5-FU tolerated over the 12 week treatment period

| *Average dose over 12 weeks (g per patient $week^{-1}$)* | *No. of patients* | *Range of equivalent doses in mg $m^{-2}$ $day^{-1}$* |
|---|---|---|
| <0.9 | 5 | 25–56 |
| 1.0–14.0 | 4 | 104–114 |
| 1.5–1.9 | 9 | 101–172 |
| 2.0–2.4 | 6 | 159–188 |
| 2.5–2.9 | 5 | 188–244 |
| 3.0–3.4 | 1 | 236 |

**Figure 1** Survival of all patients treated with 5-fluorouracil and alpha interferon ($n = 30$, median survival = 7months).

**Figure 2** Survival of patients alive 3 months after treatment by response achieved (partial response (PR) + stable disease (SD), $n = 8$; disease progression (PD), $n = 12$). Patients who died before completion of the initial trial period of 3 months were excluded ($n = 9$), as was one patient whose treatment was interrupted by surgery.

ferase, alkaline phosphatase, α-glutamyltransferase or lactate dehydrogenase values. Survival of patients with advanced and recurrent disease was similar.

*Toxicity*

The combination of 5-FU and alpha interferon was well tolerated and there were no treatment-related deaths. Table IV shows the incidence of toxic effects. Oral mucositis was common (12/30 patients), mild to moderate in severity (WHO grade 1–2) and frequently accompanied by candidiasis (5/30 patients). Overall, oral mucositis was the principal reason for treatment delay and dose reduction. Patients reporting nausea (8/30 patients) or transient diarrhoea (4/30 patients) responded to simple medication. Palmar and plantar erythema/exfoliation was uncommon (3/30 patients) and no specific treatment was required. Toxic effects attributable to alpha interferon (flu-like symptoms, fatigue and somnolence) were frequently reported at the start of treatment but diminished with time. Alpha interferon was stopped after 6 weeks in one patient because of persistent and severe symptoms. Haematological toxicity was rare. One patient developed abrupt neutropenia (WBC $0.7 \times 10^6 l^{-1}$) but completed treatment at a reduced dose after a 1 week delay. In

**Table IV** Incidence of toxic effects ($n = 30$ patients)

| | *No. of patients* WHO grade 1–2 | WHO grade 3–4 |
|---|---|---|
| Attributable to 5-FU | 12 | 0 |
| Nausea | 8 | 0 |
| Diarrhoea | 4 | 0 |
| Palmer/plantar erythema | 3 | 0 |
| Oral mucositis | 12 | 0 |
| Oral candidiasis | 5 | 0 |
| Other infection | 3 | 0 |
| Neutropenia | 0 | 1 |
| Thrombocytopenia | 0 | 1 |
| Attributable to alpha IFN | | |
| Flu-like symptoms | 10 | 1 |
| Fatigue | 5 | 1 |
| Fevers | 1 | 1 |
| Somnolence | 1 | 1 |

one patient treatment was delayed 1 week because of thrombocytopenia (platelet count $47 \times 10^{12} l^{-1}$), but treatment was subsequently completed at the same dose level without further incident. Two patients required blood transfusions for occult gastrointestinal haemorrhage. One patient required emergency surgery for a gastrocolic fistula.

### Discussion

We report a disappointingly low response rate (overall response 20%) to continuous infusional 5-FU in combination with alpha interferon in patients with metastatic colorectal carcinoma. Previous studies using these agents (continuous infusional 5-FU alone or alpha interferon and 5 day infusions of 5-FU) have reported very variable results (30–53% and 26–63% response rates respectively) (Leichman *et al.*, 1985; Wade *et al.*, 1988; Kenemy *et al.*, 1990; Wadler and Weirnik, 1990; Lokich *et al.*, 1991; Wadler *et al.*, 1991). Overall, our response rates were the lowest recorded. This apparent inconsistency may be explained in part by sampling error inherent in trials involving small numbers of patients, and by variation in tumour measurement methodology and in the degree of stringency in application of response criteria, particularly when there are liver or lung metastases. In this trial, only objective radiological criteria were used and the UICC response criteria were rigidly applied.

In direct contrast to previous studies, we elected to treat patients only when they became symptomatic. Inevitably, this included some patients with large tumour burdens and declining performance status, some of whom rapidly entered the terminal phase within a few weeks of starting treatment and died before completing the 3 month course (9/30 patients). By excluding asymptomatic patients, we may have selected a subgroup of patients with more advanced disease and a low potential for tolerating and responding to treatment. Thus, our results may not be strictly comparable to other trials which included asymptomatic and/or minimally symptomatic patients.

Two studies provide evidence for inferior responses to treatment and survival among symptomatic patients. Grem *et al.* (1991) reported a significantly lower response rate among patients with ECOG grade II symptoms (33%) than among asymptomatic or minimally symptomatic patients (53%) treated with the same regimen. The Nordic Gastrointestinal Tumour Adjuvant Therapy Group (1992) found that the median survival, asymptomatic phase and time to disease progression may be improved by as much as 6 months by early treatment of asymptomatic patients. However, treating all patients in the asymptomatic phase would be a large clinical undertaking and does not take into account the variable course of metastatic carcinoma of the colon. At one end of the disease spectrum there are patients who become symptomatic and rapidly enter the terminal phase, whereas others may be asymptomatic or minimally symptomatic for long periods of time from the diagnosis of metastases (53–621 days in our series) and continue to have a prolonged clinical course in the symptomatic phase. Clearly, it is essential to identify reliable prognostic criteria which would aid selection of those patients most likely to benefit from early treatment and those for whom expectant treatment would be more appropriate.

Two additional factors which may have compromised our response rates are the low doses of 5-FU and alpha interferon administered. The intended dose of 5-FU in most studies using single-agent infusional 5-FU is 300 $mg\,m^{-2}\,day^{-1}$, with dose reductions to 250–200 $mg\,m^{-2}\,day^{-1}$ in the event of toxic effects. Our patients commenced at 2.5 $g\,week^{-1}$, and half (15/30) required subsequent dose reduction. The majority received an average of 1.5–2.9 $g\,week^{-1}$. This dose is equivalent to 101–244 $mg\,m^{-2}\,day^{-1}$, and the average dose received (170 $mg\,m^{-2}\,day^{-1}$) is clearly well below the doses tolerated in other studies using 5-FU alone. The dose-limiting side-effect was oral mucositis and, to a lesser extent, nausea and diarrhoea.

Although WHO grade 3–4 mucositis did not occur, we found that persistent mild mucositis was poorly tolerated over the long treatment period and precluded subsequent dose escalations.

We used a fixed low dose of alpha interferon ($3 \times 10^6$U × three times a week) with the initial intent of maximising the dose of the primary active agent, 5-FU. The clinical effect of alpha interferon on colorectal carcinoma trials does not appear to be dose related. More responses have been reported with 5-FU in combination with low doses of alpha interferon compared with high doses: five out of nine patients treated with 6 or $9 \times 10^6$U responded compared with none treated with 12, 15 or $18 \times 10^6$U × three times a week (Wadler *et al.*, 1990) and 4/9 and 4/7 treated with 3 and $5 \times 10^6 U\,m^{-2}$ daily, respectively, compared with 2/6 treated at $10 \times 10^6 U\,m^{-2}$ daily (Grem *et al.*, 1991). By contrast, toxic effects appear to increase with increasing doses of alpha interferon. When compared with high doses of alpha interferon, low-dose regimens (3 or $5 \times 10^6 U\,m^{-2}$ daily) are associated with a lower incidence of severe mucositis, CNS toxicity and a lower requirement for dose reduction of 5-FU (Grem *et al.*, 1991). However, the dose of 5-FU tolerated by most of our patients (101–244 $mg\,m^{-2}\,day^{-1}$) was lower than that tolerated by others treated with single-agent continuous infusional 5-FU (200–300 $mg\,m^{-2}\,day^{-1}$), suggesting that even $3 \times 10^6$U of alpha interferon three times a week may increase the toxicity of 5-FU (Lokich *et al.*, 1989, 1991; Wadler *et al.*, 1991; Findlay *et al.*, 1993). This finding corroborates those of Meadows *et al.* (1991), who reported an increase in mucositis and a low actual dose of 5-FU delivered (160 $mg\,m^{-2}\,day^{-1}$) with doses of alpha interferon in excess of $2 \times 10^6\ U\,m^{-2}$ three times a week in patients with gastrointestinal tumours. Similarly, in a study by Grem *et al.* (1991), addition of alpha interferon reduced the maximum tolerated dose of 5-FU.

This phenomenon may be due in part to the pharmakokinetic and biochemical interactions between the two agents. At doses of 5 and $10 \times 10^6\ U\,m^{-2}\,day^{-1}$, alpha interferon increases the total exposure of 5-FU by increasing the half-life and reducing its clearance (Grem *et al.*, 1991). Additional clinical and toxic effects may be due to the synergistic biochemical interaction of 5-FU and alpha interferon on the inhibition of thymidylate synthetase which has been demonstrated in preclinical studies. A recent clinical study using magnetic resonance spectroscopy to monitor the metabolism of 5-FU provides evidence for biomodulation *in vivo*: addition of alpha interferon increased the accumulation of 5-FU in responsive tumours and was associated with the appearance of new cytotoxic anabolites not found with single-agent 5-FU (Findley *et al.*, 1993).

In this study, we found no evidence of clinically beneficial chemomodulation of 5-FU by alpha interferon. However, as the continuous infusional 5-FU was extremely well tolerated and some patients may have received suboptimal doses, we plan to further assess its efficacy as a single agent given to dose-limiting toxicity over long periods of time in symptomatic patients. As a potentially large number of patients may be eligible for treatment each year, the feasibility of community-based care is being studied in parallel.

**Acknowledgements**
It is a pleasure to acknowledge the help of David Ryder, who did the statistical analysis, and the secretarial assistance of Julie Taylor.

**References**

ANSFIELD F, KLOTZ J, NEALON T, RAMIREZ G, MINTON J, HILL G, WILSON W, DAVIS H AND CORNELL G. (1977). A phase III study comparing the clinical utility of four regimes of 5-fluorouracil. A preliminary report *Cancer*, **39**, 34–40.

AUSMAN R, CABALLERO G, QUEBBEMAN E AND HANSEN R. (1985). Response of metastatic colorectal adenocarcinomas to long term antulatory intravenous infusion (CaII) of 5 fluorouracil (5-FU) (abstract C-336). *Proc. ASCO*, **4**, 86.

ERLICHMAN C, FINE S, WONG A AND ELHAKIM T. (1988). A randomised trial of 5-fluorouracil with folinic acid in patients with metastatic colorectal carcinoma. *J. Clin. Oncol.*, **6**, 469–475.

FINDLAY MPN, LEACH MO, CUNNINGHAM D, COLLINS DJ, PAYNE GS, GLAHOLM J, MANSI JL AND McCREADY VR. (1993). The non-invasive monitoring of low dose, infusional 5-fluorouracil and its modulation by interferon-alpha using *in vivo* $^{19}F$ magnetic resonance spectroscopy in patients with colorectal cancer: pilot study. *Ann. Oncol.*, **4**, 597–603.

FORNASIERO A, DANIELE O, GHIOTTO C, AVERVSA SM, MORANDI P AND FIORENTINO MV. (1990). Alpha-2 interferon and 5-fluorouracil in advanced colorectal cancer. *Tumori*, **76**, 385–388.

GREM JL, McATEE N, MURPHY RF, BALIS FM, STEINBIERG SM, HAMILTON JM, SORENSEN JM, SARTOR O, KRAMEN BS, GOLDSTEIN LJ, GAY LM, CAUBO KM, GOLDSPIEL B AND ALLEGRA CJ. (1991). A pilot study of Interferon alfa 2a in combination with fluorouracil plus high-dose leucovorin in metastatic gastrointestinal carcinoma. *J. Clin. Oncol.*, **9**, 1811–1820.

HARTMAN HA, KESSINGER A, LEMON HM AND FOLEY JF. (1979). 5-day continuous infusion of 5-fluorouracil for advanced colorectal, gastric and pancreatic adenocarcinoma. *J. Surg. Oncol.*, **11**, 227–238.

HORTON J, OLSON KB, SULLIVAN J, REILLY C AND SCHNIDER B. (1970). 5-fluorouracil in cancer: an improved regimen. *Ann. Intern. Med.*, **73**, 897–900.

HUBERMAN M, BERING H AND TESSITORE J. (1990). 5-fluorouracil plus combined alpha interferon in advanced colorectal cancer (abstract). *Proc. Am. Soc. Clin. Oncol.*, **9**, 116.

KEMENY N, YOUNES A, SEITER K, KELSEN D, SAMMARCO P, ADAMS L, DERBY S, MURRAY P AND HOUSTON C. (1990). Interferon alpha-2a and 5-fluorouracil for advanced colorectal carcinoma. Assessment of activity and toxicity. *Cancer*, **66**, 2470–2475.

LEICHMAN L, LEICHMAN CG, KINZIE J, WEAVER D AND EVANS L. (1985). Long term low dose 5-Fluorouracil (5-FU) in advanced measurable colon cancer. No correlation between toxicity and efficacy (abstract). *Proc. Am. Soc. Clin. Oncol.*, **4**, (86).

LEICHMAN CG, LEICHMAN L, SPEARS CP, ROSEN PJ, MUGGIA F, JEFFERS S AND WAUGH W. (1990). Biological modification of protracted infusion of 5-Fluorouracil with weekly Leucovorin. *Cancer Chemother. Pharmacol.*, **26**, 57–61.

LOKICH JJ, BOTHE A, FINE N AND PERRI J. (1981). Phase I study of protracted venous infusion of 5-fluorouracil. *Cancer*, **48**, 2565–2568.

LOKICH JJ, AHLGREN JD, GULLO JJ, PHILLIPS JA AND FRYER JG. (1989). A prospective randomised comparison of continuous infusion fluorouracil with a conventional bolus schedule in metastatic colorectal carcinoma: a mid-Atlantic oncology program study. *J. Clin. Oncol.*, **7**, 425–432.

LOKICH JJ, AHLGREN JD, CANTRELL J, HEIM WJ, GALEN L, WAMPLER GL, GULLO JJ, FRYER JG AND ALT DE. (1991). A prospective randomised comparison of protracted infusional 5-fluorouracil with or without weekly bolus cis platin in metastatic colorectal carcinoma. A mid-Atlantic Oncology Program study. *Cancer*, **67**, 14–19.

MACMILLAN WE, WOLBERG WH AND WELLING PG. (1978). Pharmacokinetics of 5-fluorouracil in humans. *Cancer Res.*, **38**, 3479–3482.

MEADOWS LM, WALTHER P AND OZER H. (1991). Alpha interferon and 5-fluorouracil: possible mechanisms of antitumour action. *Semin. Oncol.*, **18**: 71–76.

NOBILE MT, VIDILLI MG, SOBRERO A, GALLIGIONI E, FASSIO T, LO-REG RUBAGOTTI A, SERTOLI MR AND ROSSO R. (1988). A randomised trial of 5-fluorouracil and high dose leucovorin in untreated advanced colorectal cancer patients. *Adv. Exp. Med. Biol.*, **244**, 213–218.

NORDIC GASTROINTESTINAL TUMOUR ADJUVANT THERAPY GROUP. (1992). Expectancy of primary chemotherapy in patients with advanced asymptomatic colorectal cancer: a randomised trial. *J. Clin. Oncol.*, **6**, 904–911.

OPCS. (1986). Cancer Statistics, Series MB1, Vol. 19, Table 2. OPCS Publications: London.

PAZDUR R, AJANI JA, PATT YZ, WINN R, JACKSON D, SHEPARD B, DUBRO R, CAMPOS L, QUARAISHI M, FAINTUCH J, ABBRUZZESE JL, GUTTERMAN J AND LEVIN B. (1990. Phase II study of fluorouracil and recombinant interferon alfa-2a in previously untreated advanced colorectal carcinoma. *J. Clin. Oncol.*, **8**, 2027–2031.

PETRELLI N, STABLEIN D, BRUCKNER H, MEGIBOW A, MAYER R AND DOUGLASS H. (1988). A prospective randomised phase III trial of 5-fluorouracil (5-FU) versus 5-FU plus high dose leucovorin (HDCF) versus 5-FU plus low dose leucovorin (LDCF) in patients (pts) with metastatic colorectal adenocarcinoma. A report of the GI tumour study group (abstract). Proc. Am. Soc. Clin. Oncol., **7**, 94.

POPLIN EA, KRAUT M, BAKER L, BRODFUEHRER J AND VAITKEVICIUS V. (1991). A dose intensive regime of 5-fluorouracil for the treatment of metastatic colorectal carcinoma. *Cancer*, **67**, 367–371.

SIEFERT P, BAKER LH, REED ML AND VAITKEVICIUS V. (1975). Comparison of continuously infused 5-fluorouracil with bolus injection in treatment of patients with colorectal adenocarcinoma. *Cancer*, **35**, 123–128.

ULLMAN B, LEE M, MARTIN DW Jr. AND SANTI DV. (1978). Cytotoxicity of 5-Fluoro-2-deoxyuridine: requirement for reduced folate co-factors and antagonism by methotrexate. *Proc. Natl Acad. Sci. USA*, **75**, 980–893.

WADE JL, HERBST S AND GREENBERG A. (1988). A prolonged venous infusion (PVI) of 5-fluorouracil (5FU) for metastatic colon cancer (MCC) – a follow up report (abstract). *Proc. Am. Soc. Clin. Oncol.*, **7**, 94.

WADLER SW AND WIERNIK PH. (1990). Fluorouracil and recombinant alfa-2a-interferon: an active regimen against advanced colorectal carcinoma. *J. Clin. Oncol.*, **7**, 1769–1775.

WADLER S, WERSTO R, WEINBERG VE, THOMPSON D AND SCHWARTZ EL. (1990). Interactions of fluorouracil and interferon in human colon cancer cell lines. Cytotoxic and cytokinetic effects. *Cancer Res.*, **50**, 5735–5739.

WADLER S, LEMBERSKY B, ATKINS M, KIRKWOOD J AND PETRELLI N. (1991). Phase II Trial of fluorouracil and recombinant Interferon alfa 2a in patients with advanced colorectal carcinoma. an Eastern co-operative Oncology Group Study. *J. Clin. Oncol.*, **9**, 1806–1810.